# Anaesthetic-dependent changes in gene expression following acute and chronic exposure in the rodent brain

**DOI:** 10.1038/s41598-020-66122-6

**Published:** 2020-06-09

**Authors:** Dannielle H. Upton, Kata Popovic, Roger Fulton, Michael Kassiou

**Affiliations:** 1 Faculty of Medicine and Health, The University of Sydney, Sydney, NSW Australia; 2 School of Chemistry, The University of Sydney, Sydney, NSW Australia; 3 Department of Medical Physics, Westmead Hospital, Sydney, Australia

**Keywords:** RNA, Molecular neuroscience

## Abstract

Anaesthesia has been predicted to affect gene expression of the memory-related regions of the brain including the primary visual cortex. It is also believed that anaesthesia causes inflammation of neural tissues, increasing elderly patients’ chances of developing precursor lesions that lead to Alzheimer’s disease and other neurodegeneration related diseases. We have analyzed the expression of over 22,000 genes and 129,800 transcripts using oligonucleotide microarrays to examine the brain expression profiles in Sprague Dawley rats following exposure to acute or chronic doses of the anaesthetics isoflurane, ketamine and propofol. Here we report for the first time molecular and genomic data on the effect on the rodent brain of chronic and acute exposure to isoflurane, ketamine and propofol. Our screen identified multiple genes that responded to all three anaesthetics. Although some of the genes were previously known to be anaesthesia responsive, we have for the most part identified novel genes involved in the acute and chronic rodent brain response to different anaesthesia treatments. The latter may be useful candidate genes in the search to elucidate the molecular pathways mediating anaesthetic effects in the brain and may allow us to identify mechanisms by which anaesthetics could impact on neurodegeneration.

## Introduction

Volatile anaesthetics have been in use for about 170 years and are used for millions of surgical procedures in humans and animals every year^1^. Anesthetics are administered to patients and animals with the aim of reducing the effect of external stimuli, providing amnesia and unresponsiveness. Anesthesia also induces muscle relaxation and makes the patient lie still, which aids the surgeon in their work. They have many modulatory effects on neuronal ionophores, but it is not clear that these changes in membrane potential and current flow are the exclusive mechanisms of anesthetic action^2^. To minimize risks, the effects of anaesthetics on the neurological system need to be understood. Currently used anesthetics are believed to act by two main mechanisms: (a) an increase in inhibition via GABA_A_ receptors (e.g. benzodiazepines, barbiturates, propofol, etomidate, isoflurane, enflurane and halothane)^3^ or (b) a decrease in excitation through NDMA receptors (e.g. ketamine, nitrous oxide and xenon)^4,5^.

Although mechanisms of action have been proposed for anaesthetics such as isoflurane, ketamine and propofol, very few studies have examined the effects of these anaesthetics on rat brain gene expression or the relationship between anaesthesia and neurodegeneration. A study by Edmands *et al*.^6^ examined the effect of acute isoflurane exposure on liver, kidney and heart tissues. They identified 34 genes that play roles in regulation of inflammation, modulation of apoptosis, regulation of ion gradients and maintenance of energy pathways and were potentially clinically relevant in understanding how anesthetic exposures can lead to protection against ischemic injury. This study did not examine the effect of acute isoflurane on brain tissue. Another study by Bunting *et al*.^7^ assessed whether general anesthesia with isoflurane stops transcription initiated prior to anesthetic administration. They found that on a cellular level, isoflurane administered at high doses (general anesthesia) prevented initiation of transcription but did not stop *Arc* and *Zif268* mRNA transcription initiated prior to anesthesia. They suggest that different levels of anesthesia affect memory via different mechanisms with general anesthesia preventing elevation of mRNA levels of *Arc* and *Zif268* necessary for normal memory formation, while lower dose anesthesia affects the strength of memory by altering levels of plasticity-related proteins. This study only looked at the effects of acute isoflurane exposure and mRNA levels were quantified from *in situ* hybridization methods rather than microarray analysis.

A study by Liu *et al*. assessed the effects of ketamine administration to postnatal day 7 rat pups, focusing on potential dose- and time-dependent neurotoxic effects and associated changes in gene expression. This study suggested that recurrent exposures to high doses of ketamine can cause compensatory up-regulation of NMDA receptors (by microarray analysis and RT-PCR confirmation) and subsequently trigger apoptosis in developing neurons. Two similar studies by Goto *et al*.^8^ and Lu *et al*.^9^ examined the effects of propofol and sevoflurane on hippocampal miRNA expression. Goto *et al*. demonstrated that sevoflurane and propofol anesthesia induced several changes in the miRNA expression levels of the rat hippocampus. Both studies focused on the hippocampus alone, acute anaesthetic exposure, and used an extraction protocol and array focused only on miRNA and not RNA expression changes.

Pan *et al*.^10^ observed that sevoflurane induced long-term (at least 2 days) expression change of numerous hippocampal genes, which may be related to memory impairment or other neural disorders. This study however focused only on acute sevoflurane exposure and only examined the hippocampus of the exposed animals. Another study by Wang *et al*.^11^ examined the effects of halothane on the brain tissue of rats. They demonstrated that halothane modulated the expression of 44 differentially expressed genes, which were involved predominantly in responses to endogenous and corticosteroid stimuli. Although this study did look at both chronic and acute exposure on anesthetics on whole brain, it only examined halothane.

Very few studies have examined the effects of acute and chronic anaesthetic exposure in relation to neurodegeneration. Some studies have been undertaken on neonatal and postnatal exposure to isoflurane^12,13^, ketamine^14–17^ and propofol^18–20^, however none focus on acute vs chronic exposure or whole brain gene expression changes. A study by Kalenka *et al*.^21^ examined changes in the hippocampal proteome of rats post isoflurane exposure. Using differential 2-dimensional electrophoresis, mass spectrometry, and functional network mapping they identified and functionally classified 12 different hippocampal proteins, which were significantly regulated after isoflurane anaesthesia. According to their key biologic activities, the differentially expressed proteins were grouped into categories such as synaptic plasticity, stress response, detoxification, and cytoskeleton in early and late recovery phases after anaesthesia which showed that isoflurane affects these biologic processes which also are affected in Alzheimer’s disease^21^. A study by Liu *et al*.^22^ examined the effects of single and repeated doses of ketamine on post-natal rats. They found that repeated exposures to high doses of ketamine can cause compensatory up-regulation of NMDA receptors and subsequently trigger apoptosis in developing neurons^22^.

Here we report for the first time molecular and genomic data on the effect on the rodent brain of chronic and acute exposure to isoflurane, ketamine and propofol. We hypothesized that with RNA microarray analysis of gene expression in anaesthetic-treated and control rats we would be able to identify genes that were regulated by the different anaesthetic regimens. This has allowed us to identify mechanisms by which anaesthetics could impact on neurodegeneration. Our aim was to gain an insight into the molecular mechanisms underlying first episode anaesthetic effects and the effect of repeated anaesthetic exposure, including the identification of genes that may explain individual-specific responses to anaesthesia and also potentially identify mechanisms in which anaesthetics affect neurodegeneration.

## Results

### Measured parameters during treatments

Respiration rate was measured in treated animals (not controls due to free mobility within the treatment chamber) and no significant difference was observed between isoflurane (69 ± 15 breaths per minute (bpm)), ketamine (66 ± 25 bpm) or propofol (76 ± 11 bpm) treatments (data not shown). Anaesthetic induction time and recovery time was measured in the treated animals. Induction time was similar in both the Isoflurane (178 ± 37 seconds) and Ketamine (175 ± 56 seconds) treated groups (p = 0.9) but was significantly longer and more variable in the propofol treated group (823 ± 464 seconds, isoflurane vs propofol p = 0.004; ketamine vs propofol p = 0.006)(data not shown). Isoflurane treated animals had a faster recovery rate (248 ± 127 seconds; isoflurane vs ketamine p = 0.001; isoflurane vs propofol p = 0.06), whilst ketamine and propofol treated animals had a longer and more variable recovery rate (2856 ± 1472 seconds and 1254 ± 1296 seconds respectively; p = 0.1)(data not shown).

### Brain weight

Average brain weight of both acute and chronic treated rats were not statistically significantly different between groups; acute control 2.08 ± 0.11 g, acute isoflurane 2.0 ± 0.07 g, acute ketamine 2.02 ± 0.08 g, acute propofol 2.08 ± 0.11 g, chronic control 2.13 ± 0.10 g, chronic isoflurane 2.11 ± 0.14 g, chronic ketamine 2.05 ± 0.14 g and chronic propofol 2.10 ± 0.09 g (one-way ANOVA, acute *p* = 0.35; chronic *p* = 0.60).

### Microarray gene expression changes between acute and chronic treatments

Gene expression changes were compared between acute and chronic control, isoflurane, propofol and ketamine treatment groups. Comparing acute control and chronic control treated animals, a total of 18 genes showed more than 2-fold change in gene expression, 6 of which (33%) were up-regulated and 12 (67%) were down-regulated (Table 1). *Fcrl2, Slamf1, Tshb, Olr1700* and *Cyp4v3* were the most significantly increased whilst *Tph1, Pde6g, Neurod4, Gnat* and *Pde6c* were found to be the most significantly decreased (Supplementary Table 1).

**Table 1:** Summary of biostatistical and bioinformatic analyses for individual treatments – Acute Control vs Acute treatment and Chronic Control vs Chronic Treatment. GO – gene ontology terms.

	Acute v Chronic				Acute treatment v Acute Control			Chronic treatment v Chronic Control		
	Control	Isoflurane	Ketamine	Propofol	Isoflurane	Ketamine	Propofol	Isoflurane	Ketamine	Propofol
Total Significant genes (% of genes examined)	18 (0.08%)	17 (0.07%)	9 (0.04%)	5 (0.02%)	27 (0.12%)	34 (0.15%)	24 (0.1%)	18 (0.08%)	18 (0.08%)	15 (0.06%)
Significant Increase (% significant genes)	6 (33.33%)	12 (70.59%) 11 coding, 1 unassigned	4 (44.44%)	2 (40%)	16 (59.26%) 14 coding, 1 non-coding, 1 unassigned	16 (47.06%) 16 coding	14 (58.33%)	9 (50%) 8 coding, 1 non-coding	7 (38.89%) 7 coding	6 (40%) 4 coding, 2 multi complex
Significant decrease (% significant genes)	12 (66.67%)	5 (29.41%), 5 coding	5 (55.56%)	3 (60%)	11 (40.74%) 11 coding	18 (52.94%) 17 coding, 1 non-coding	10 (41.67%)	9 (50%) 9 coding	11 (61.11%) 11 coding	9 (60%) 9 coding
Maximum significant fold increase	4.01	246.3	2.35	2.47	4.86	4.75	6.13	3.14	3.03	4.7
Maximum significant fold decrease	258.71	2.28	2.4	2.14	230.12	125.39	259.24	4.37	4.39	19.01
Significant Functional GO categories	11	1	0	0	26	10	20	64	7	11

The significantly altered genes can be divided into 11 clusters including phototransduction, visual perception, retinal cone cell development, mammary gland alveolus development, positive regulation of B cell activation, phagocytosis (recognition), phagocytosis (engulfment), complement activation (classical pathway), B cell receptor signalling pathway, G-protein couple receptor signalling pathway and embryonic skeletal system morphogenesis (data not shown).

Comparing gene expression changes between our acute isoflurane and chronic isoflurane treated animals, a total of 17 genes showed a more than 2-fold change in gene expression, of which 12 (71%) were up-regulated and 5 (29%) were down-regulated (Table 1). *Asb15, RT1-M6-2, Wdr49, Frmd7* and *Grifin* were the most significantly increased whilst *Plekhh3, Nuak2, Ttc30a, XAF1* and *C3* were found to be the most significantly decreased (Supplementary Table 1). Two genes (*Lmod2, LOC100909784)* were found to cluster into the same gene ontology term of pointed-end actin filament capping (data not shown).

When comparing gene expression changes between acute ketamine and chronic ketamine treated animals, 9 genes showed more than 2-fold change in gene expression, of which 4 (44%) were up-regulated and 5 (56%) were down-regulated (Table 1). *Ly49s7, Ifna16l1, Olr586* and *Vom2r1* were the most significantly increased whilst *Sycp2l, Avp, Mcm5, Bpifb3* and *Inca1* were found to be the most significantly decreased (Supplementary Table 1).

Comparing gene expression changes between acute propofol and chronic propofol treated animals, 5 genes showed more than 2-fold change in gene expression, 2 of which (40%) were up-regulated and 3 (60%) were down-regulated (Table 1). *Bmp4* and *Klkb1* were the most significantly increased whilst *Hoxa6, Zfp72* and LO*C102553524* were found to be the most significantly decreased (Supplementary Table 1).

### Microarray gene expression changes after Isoflurane treatment

#### Acute

**Figure 1:**
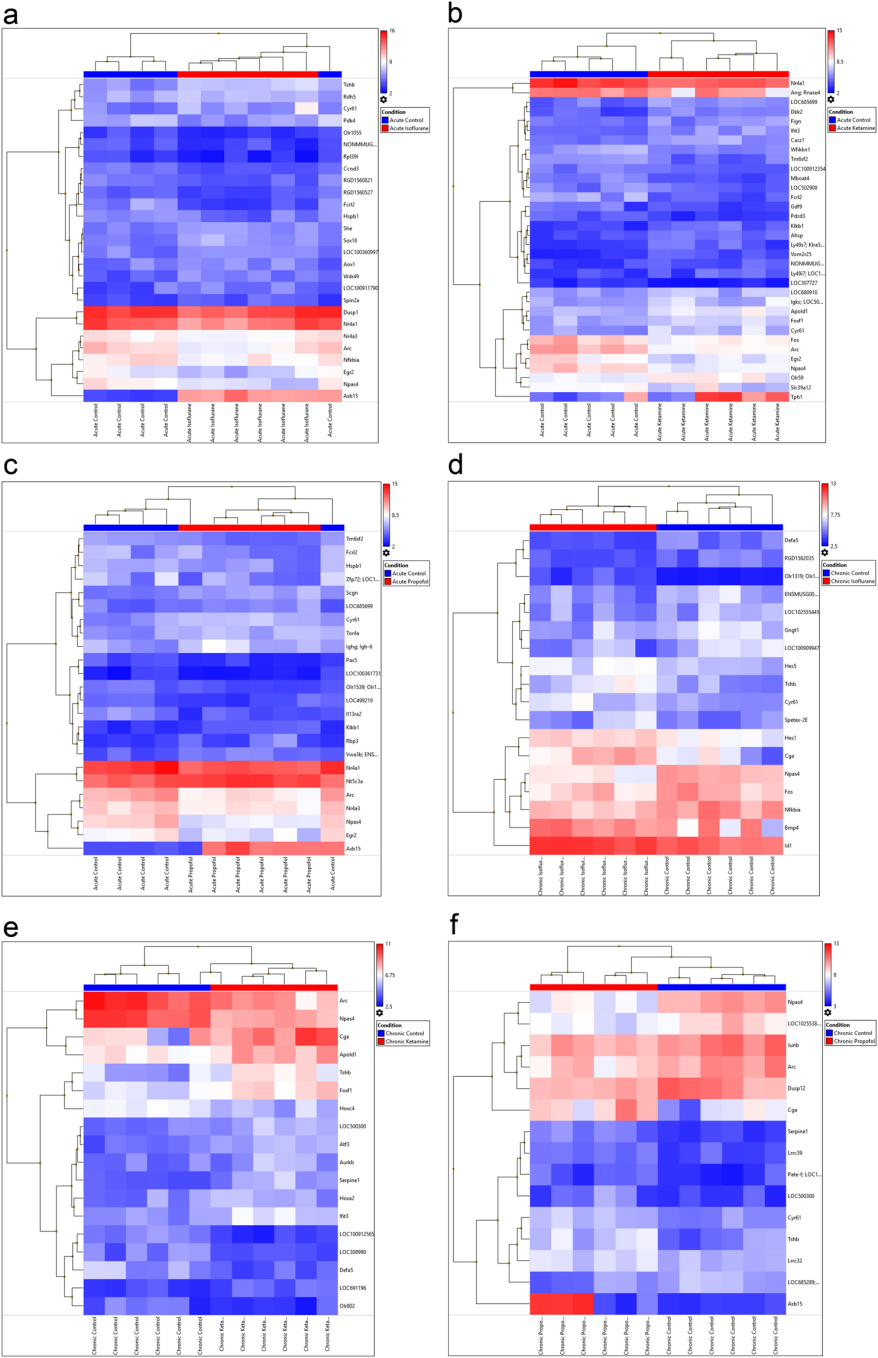
Heatmap of Treatment comparisons – Log2 average gene expression for each sample compared to appropriate control. The top horizontal axis of the image depicts colour bars indicating the grouping into either control or treatment and shows a hierarchical grouping above the colour coding, the bottom horizontal axis labels the identity of the samples, the right vertical axis labels the genes examined and the left vertical axis clusters the genes into families. The colour scale on the top right of the image allocates a colour based on the scale for each independent value per sample dictating the different gene expression level for every sample. **(a)** Acute control vs acute isoflurane**; (b)** acute control vs acute ketamine**; (c)** acute control vs acute propofol**; (d)** chronic control vs chronic isoflurane**; (e)** chronic control vs chronic ketamine**; (f)** Chronic control vs chronic propofol.

**Figure 2:**
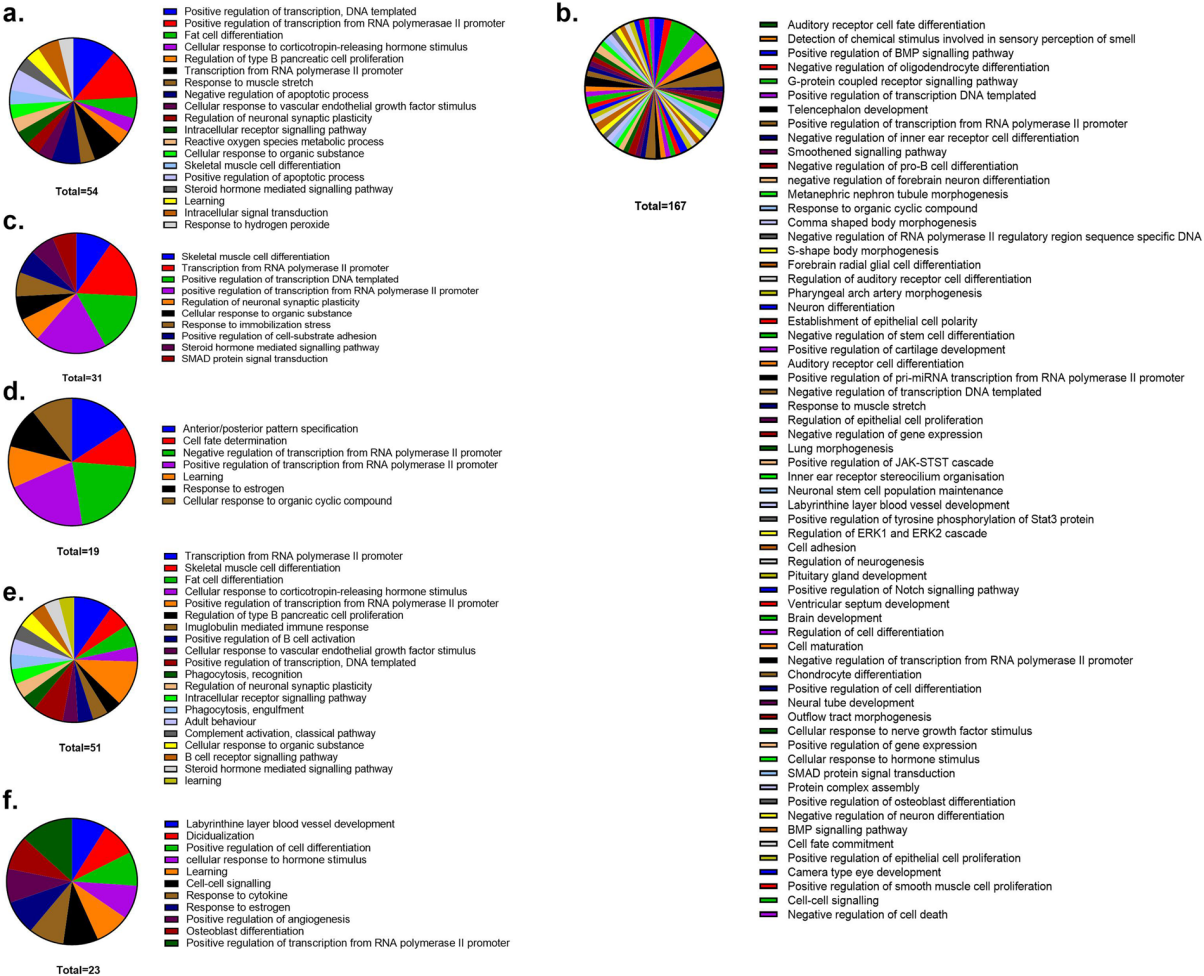
GO term clustering for control vs treatments. (**a**) Acute control vs. Acute Isoflurane – 54 genes grouped into 19 clusters; **(b)** Chronic control vs. Chronic Isoflurane – 167 genes grouped into 64 clusters; **(c)** Acute control vs. Acute Ketamine – 31 genes grouped into 10 clusters; **(d)** Chronic control vs. Chronic Ketamine – 19 genes grouped into 7 clusters; **(e)** Acute control vs. Acute propofol – 51 genes grouped into 20 clusters; **(f)** Chronic control vs. Chronic propofol – 23 genes grouped into 11 clusters.

Gene expression changes were compared between our acute control and acute isoflurane treated animals. A total of 27 genes showed more than 2-fold change in gene expression, 16 (59.26%) were up-regulated and 11 (40.74%) were down-regulated (Table 1). Figure 1a shows a heatmap of the average log2 gene expression for the comparison of acute control and acute isoflurane. *Fcrl2, Arc, Rpl39l, Npas4* and *Egr2* were found to be the 5 most significantly increased genes when comparing control to acute isoflurane treatment. *Asb15, Tshb, Rdh5, She* and *Sox18* were the 5 most significantly decreased genes when comparing control to acute isoflurane (Supplementary Table 2). The significantly altered genes can be divided into 19 clusters (Fig. 2a).

#### Chronic

Gene expression changes were compared between our chronic control and chronic isoflurane treated animals. A total of 18 genes showed more than 2-fold change in gene expression, 9 (50%) were up-regulated and 9 (50%) were down-regulated (Table 1). Figure 1d shows a heatmap of the average log2 gene expression for the comparison of chronic control and chronic isoflurane. *Npas4, Gngt1, Fos, Nfkbia* and *Defa5* expression was the most significantly increased when comparing control to chronic isoflurane. *Cga, Tshb, Bmp4, Cyr61* and *Hes5* expression were the most significantly decreased genes when comparing control to chronic isoflurane (Fig. 1d, Supplementary Table 3). The significantly altered genes can be divided into 64 clusters (Fig. 2b).

### Microarray gene expression changes after Ketamine treatment

#### Acute

Gene expression changes were compared between our acute control and acute ketamine treated animals. A total of 34 genes showed more than 2-fold change in gene expression, 16 (47%) were up-regulated and 18 (53%) were down-regulated (Table 1). Figure 1b shows a heatmap of the average log2 gene expression for the comparison of acute control and acute ketamine. *Fcrl2, Arc and Slc39a12* expression was the 3 most significantly when comparing control to acute ketamine. *Tph1, Cyr61* and *Vom2r25* expression was the 3 most significantly decreased when comparing control to acute ketamine (Fig. 1b, Supplementary Table 2). The significantly altered genes can be divided into 10 clusters (Fig. 2c).

#### Chronic

Gene expression changes were compared between chronic Control and chronic Ketamine treated animals. A total of 18 genes showed more than 2-fold change in gene expression, 7 (39%) were up-regulated and 11 (61%) were down-regulated (Table 1). Figure 1e shows a heatmap of the average log2 gene expression for the comparison of chronic control and chronic ketamine. *Npas4, Arc* and *LOC308990* expression were significantly increased when comparing control to chronic ketamine. *Tshb, Cga* and *Hoxa2* expression were the most significantly decreased when comparing control to chronic ketamine (Fig. 1e, Supplementary Table 3). The significantly altered genes can be divided into 7 clusters (Fig. 2d).

### Microarray gene expression changes after propofol treatment

#### Acute

Gene expression changes were compared between our acute Control and Acute propofol treated animals. A total of 24 genes showed more than 2-fold change in gene expression, 14 (58%) were up-regulated and 10 (42%) were down-regulated (Table 1). Figure 1c shows a heatmap of the average log2 gene expression for the comparison of acute control and acute propofol. *Zfp72, Fcrl2* and *Arc* expression were the most significantly increased when comparing control to acute propofol. *Asb15, Cyr61* and *Nt5c3a* expression were the most significantly decreased when comparing control to acute propofol (Fig. 1c, Supplementary Table 2). The significantly altered genes can be divided into 20 clusters (Fig. 2e).

#### Chronic

Gene expression changes were compared between our chronic Control and chronic propofol treated animals. A total of 15 genes showed more than 2-fold change in gene expression, 6 (40%) were up-regulated and 9 (60%) were down-regulated (Table 1). Figure 1f shows a heatmap of the average log2 gene expression for the comparison of chronic control and chronic propofol. *Npas4, Arc, and Dusp12* expression were the most significantly increased when comparing control to chronic propofol. *Asb15, Tshb and Cga* expression were the most significantly decreased when comparing control to chronic (Fig. 1, Supplementary Table 3). The significantly altered genes can be divided into 11 clusters (Fig. 2f).

### Significantly altered genes common to acute anaesthetic treatments

**Figure 3:**
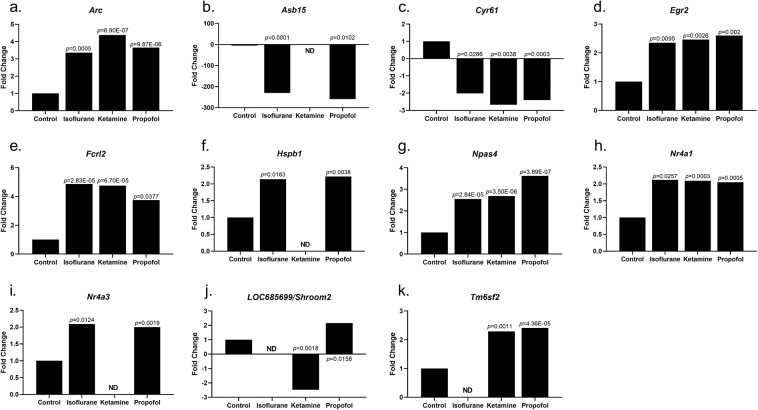
Fold change gene expression changes for Acute treatment compared to Acute control, ND – not detected **(a)** *Arc****;*** (**b**) *Asb15****;*** (**c**) *Cyr61***; (d)** *Egr2****;*** **(e)** *Fcrl2***; (f)** *Hspb1***; (g)** *Npas4***; (h)** *Nr4a1****;*** **(i)** *Nr4a3***; (j)** *LOC685699/Shroom****;*** (**k**) *Tm6sf2*.

Acute anaesthetic treatment significantly increased 6 common genes among the three anaesthetics examined when compared to controls. *Arc*, *Egr2, Fcrl2, Npas4* and *Nr4a1* were all significantly increased, while *Cyr61* was significantly decreased by 2-fold with all 3 anaesthetics examined (Table 2, Fig. 3). Ketamine and propofol significantly altered 2 genes in common, *LOC685699/Shroom* and *Tm6sf2*, when compared to control treatment (Table 2, Fig. 3). Isoflurane and propofol significantly altered 3 genes in common, *Asb15, Hspb1 and Nr4a3*, when compared to control treatment (Table 2, Fig. 3).

**Table 2:** Summary of genes significantly altered by 2 or more anaesthetic treatments.

Gene	Isoflurane	Ketamine	Propofol
***Acute anaesthetic treatment***			
*Arc*	3.36	4.38	3.65
*Asb15*	−230.12		−259.24
*Cyr61*	−2.01	−2.67	−2.4
*Egr2*	2.35	2.46	2.6
*Fcrl2*	4.86	4.75	3.75
*Hspb1*	2.14		2.22
*Npas4*	2.55	2.69	3.62
*Nr4a1*	2.12	2.09	2.05
*Nr4a3*	2.09		2
*LOC685699/Shroom2*		−2.47	2.15
*Tm6sf2*		2.29	2.41
***Chronic anaesthetic treatment***			
*Arc*		2.74	2.32
*Cga*	−4.37	−3.26	−3.18
*Cyr61*	−2.7		−2.18
*Defa5*	2.02	2.29	
*Npas4*	2.39	3.03	4.7
*Serpine1*		−2.39	−2.48
*Tshb*	−4.35	−4.39	−3.36

Data displayed as fold change/increase or decrease, common genes between both Acute and Chronic treatments.

### Significantly altered genes common to chronic anaesthetic treatments

**Figure 4:**
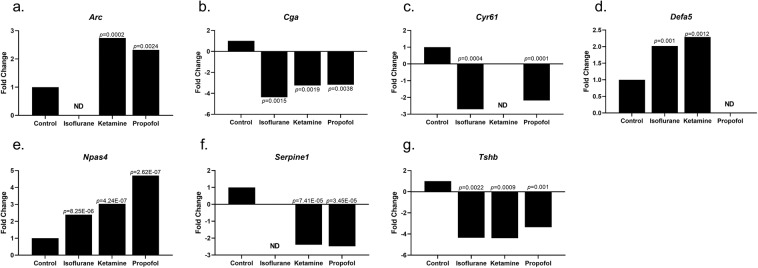
Fold change gene expression changes for Chronic treatment compared to Chronic control, ND – not detected **(a)**. *Arc****;*** (**b**). *Cga****;*** (**c**) *Cyr61****;*** (**d**) *Defa5****;*** (**e**) *Npas4***; (f)** *Serpine1****;*** **(g)** *Tsh*.

Chronic anaesthetic treatment significantly altered 3 common genes among the all three anaesthetics examined when compared to controls. Both *Cga* and *Tshb* were significantly decreased whilst *Npas4* was significantly increased when compared to controls with all 3 anaesthetic treatments (Table 2, Fig. 4). Isoflurane and ketamine significantly altered 1 common gene when compared to controls. *Defa5* was significantly increased when compared to controls (Table 2, Fig. 4). Both isoflurane and propofol significantly altered 1 common gene, *Cyr61*, which was significantly decreased when compared to controls (Table 2, Fig. 4). Ketamine and propofol significantly altered 2 genes in common, when compared to control treatment. *Arc* was significantly increased whilst *Serpine1* was significantly decreased when compared to controls (Table 2, Fig. 4).

## Discussion

Studies have previously shown that these three anaesthetics can cause widespread neuronal apoptosis in immature rat brain when administered during synaptogenesis^23,24^ but little research has been undertaken into the effects of acute and chronic anaesthesia on neurodegeneration. Several studies have found that exposure to general anaesthetics may alter neurobiological processes by inducing apoptosis and Aβ formation^25,26^. Furthermore, both preclinical and clinical studies have recently supported the notion that anaesthesia has a significant impact on tangle formation^27–29^. If indeed an increased risk of neurodegeneration were associated with anaesthesia, this would be of considerable societal significance.

Isoflurane is a general inhalation anesthetic used for induction and maintenance of anesthesia. It does this by decreasing the extent of gap junction mediated cell-cell coupling and altering the activity of action potential channels^2^. Isoflurane binds to the GABA, glutamate and glycine receptors and the large conductance Ca2+ activated potassium channel^30^. *In vitro* models of neurodegeneration have shown that isoflurane^31^, isoflurane in combination with nitrous oxide^32^, sevoflurane^33^, and desflurane with hypoxia^34^ induce apoptosis and increase Aβ formation. Another study showed in mice that exposure to isoflurane for a period of 2 hours increased neocortical and hippocampal expression of the apoptotic marker caspase 3 six hours post exposure, and elevated Aβ at 24 hours post exposure^35^. Volatile anaesthetics can increase Aβ aggregation *in vitro*^25,36^, providing an additional potential interaction between Alzheimer’s disease pathology and anaesthesia.

Ketamine is a rapid-acting general anesthetic. Ketamine produces an anesthetic state characterized by deep analgesia, normal pharyngeal-laryngeal reflexes, normal to enhanced skeletal muscle tone, cardiovascular and respiratory stimulation, and occasionally a transient and minimal respiratory depression^37^. It interacts with N-methyl-D-aspartate (NMDA), opioid, monoaminergic and muscarinic receptors, along with voltage sensitive Ca ion channels but does not interact with GABA receptors^37,38^. Ketamine is the most common anesthesia used in pediatric practice, acting primarily through blockade of NMDA-type glutamate receptors, providing sedation/analgesia to patients during proceedures^39^. Recent research has shown accelerated programmed apoptosis of nerve cells when administered with ketamine in high doses and/or for prolonged periods in rats and monkeys^40–47^.

Propofol is an intravenous anaesthetic agent used for induction and maintenance of general anaesthesia. Propofol-induced anaesthesia is associated with less frequent side effects and a more rapid recovery when compared to other intravenous anaesthetics. Propofol acts to positively modulate the inhibitory function of the neurotransmitter GABA through GABA-A receptors^48^. Propofol has proven positive attributes such as rapid onset of anaesthesia and short recovery time^49,50^. Previous studies have revealed multiple mechanisms of action, which are both age and dose dependent^18^ and suggest that propofol may protect the brain against ischemic injury^51–53^. Propofols neuroprotective effects are credited to its antioxidant properties, exponentiation of GABA_A_-mediated inhibition of synaptic transmission, and glutamate release^18,54–56^.

In recent studies, it has been shown that volatile anesthetics can induce changes in gene expression in the lung^57–59^ and in the liver^60^. In contrast to this, little is known about the action of volatile anesthetics on gene or protein expression in the brain. A previous study has shown that sevoflurane and propofol anaesthesia causes changes in brain miRNA expression patterns, indicating differential regulation of protein expression^61^. Propofol and isoflurane were also found to alter gene expression of c-fos and c-jun mRNA in a tissue dependent manner when rat heart, kidney, liver and brains were examined^62^.

Expression of apoptotic related genes was altered and NMDA receptor expression increased after repeated exposures to high doses of ketamine suggesting compensatory up-regulation of NMDA receptors and induces apoptosis in developing neurons^22^.

Our study revealed some new roles of genes that were previously unreported and was also able to link some genes that have previously been upregulated or downregulated in relation to memory, cognition, neuroprotection and learning. These included *Arc, Egr2, Hsp27, Npas4, Nr4a1, Nr4a2, Cga* and *Serpine1*. Brain-derived neurotrophic factor (BDNF) is believed to be an important regulator of striatal neuron survival, differentiation, and plasticity. BDNF has been found to play an important role in the formation, retention and recall of spatial memory^63^. BDNF plays a critical role in neuronal survival in Huntington’s Disease. The BDNF receptor is downregulated in brains from Huntington’s Disease patients when compared to unaffected healthy people^64^. Although we did not see significant changes in BDNF, we did see significantly increased expression of genes known to be induced by the neuroprotective factor BDNF^65^, including *Egr2* and *Arc*. Anaesthetic treatment significantly increased the expression of *Egr2* which is known to play a role in the BDNF pathway and the Dopamine D1R pathway. Dopamine afferents to the striatum also express BDNF, which activates TRKB receptors^66^, suggesting that a possible interaction between dopamine afferents and BDNF/TRKB signalling exists. *Egr2* is known to be crucial for normal hindbrain development and has been implicated in several inherited peripheral neuropathies^67^. *Egr2* regulates peripheral nerve myelination^68^, hindbrain segmentation^69,70^ and endochondreal bone formation^71^. *Egr2*-deficient mice display no sign of locomotor, exploratory or anxiety disturbances and no impairment in spatial learning and memory, taste aversion or fear memory^67^. *Egr2*-deficient mice have improved performance in motor learning on a rotarod, and in object recognition memory^67,72^. Our study found a significant increase in *Egr2* expression in animals treated acutely with isoflurane, ketamine and propofol. *Arc*, in mammals, is required for protein synthesis-dependent forms of long-term potentiation and depression^73,74^. *Arc* regulates synaptic plasticity through the trafficking of AMPA-type glutamate receptors (AMPARs) via the endocytic machinery^75^. This endocytic pathway maintains levels of surface AMPARs in response to chronic changes in neuronal activity through synaptic scaling, thus contributing to neuronal homeostasis^76^. Arc is required *in vivo* for long-term memory^77,78^ and to transduce experience into long-lasting changes in visual cortex plasticity^79^. *Arc* RNA expression levels in rats, induced by learning has been found to be important in memory consolidation processes^80^. In addition, *Arc* has been implicated in neurodevelopmental disorders, such as Angelman^81,82^ and Fragile X syndrome^83^, and schizophrenia^84–86^ and various neurological disorders including Alzheimer’s disease^87^. Because of this, specific regulation of nervous system *Arc* expression and activity appears to be vital for normal cognition. A study by Wu *et al*.^87^ suggested that the functions of *Arc* in both neural plasticity and Aβ generation, proposes a link between these processes that is altered in Alzheimer’s disease. Patients with Alzheimer’s disease can express high levels of Arc, and it is suggested that *Arc* participates in the pathogenesis of Alzheimer’s disease^87^. Greer *et al*.^81^ showed that disruption of *Ube3A* function in neurons promotes increased *Arc* expression and an associated decrease in the number of AMPA receptors at excitatory synapses which may contribute to cognitive dysfunction. Our study found increased levels of *Arc* in rodents treated acutely with isoflurane, ketamine and propofol or chronically with ketamine and propofol. This suggests that both chronic and acute anesthesia increases *Arc* expression and may downstream lead to the development of disorders such as Alzheimer’s disease^87^ or Angelman Syndrome and possibly other Autism Spectrum Disorders^81^.

We found 3 genes that play important roles in learning and memory with altered expression in response to anaesthesia including *Npas4, Nr4a1* and *Nr4a2*. The neuronal Per-Arnt-Sim domain protein 4 (*Npas4*) is a transcriptional regulator of synaptic plasticity and cognition^88^. *Npas4*, an activity-dependent transcription factor, regulates the transcription of discrete genes and transcriptionally controls experience-dependent learning and memory. Most recently, contextual fear experience in mice induces *Npas4* in the hippocampus, contributing to contextual memory formation and fear memory^89,90^. *Npas4*, in the striatum, is sensitive to psychostimulant exposure. A rapid and transient increase in *Npas4* mRNA in the mouse striatum was induced by an acute injection of cocaine^91,92^ suggesting that *Npas4* is a drug-responsive gene and may be involved in the regulation of drug effects. Our study found significantly increased *Npas4* expression in rats treated both acutely and chronically with isoflurane, ketamine and propofol. Previously it has been shown that expression of both *Nr4a1* and *Nr4a2* increases in the hippocampus following learning^93^. *Nr4a1* and *Nr4a2* expression increases in the hippocampus during context shock memory consolidation and following a spatial discrimination task^94–97^. *Nr4a1* is also known to be upregulated by seizure activity^98^. Another study found that a single injection of cocaine or morphine in rats leads to activation of NGFI-B and *Nr4a3/Nor1* mRNA^99^. We found significantly increased expression of *Nr4a1* in rats acutely treated with isoflurane, ketamine and propofol, however significantly increased gene expression of *Nr4a3* was only seen in isoflurane and propofol acutely treated animals.

We found two genes related to neuroprotection that were significantly altered in response to anaesthesia including *Hsp27* and *Serpine1*. Heat shock proteins (Hsps) are highly conserved proteins that are stimulated in response to both physiological and environmental stressors. *HspB1* (*Hsp27*) is strongly induced during the stress response and increases cell survival when exposed to cytotoxic stimuli by exerting its neuroprotective effects^100^. HspB1 is an ATP-independent chaperone, functioning especially in protection against protein aggregation^101^. Our study found significantly increased expression of *HspB1* in animals treated acutely with isoflurane and propofol (but not ketamine). Plasminogen activator inhibitor-1 (PAI-1), also known as endothelial plasminogen activator inhibitor or serpin E1, is a protein that in humans is encoded by the SERPINE1 gene. *Serpine1* is expressed after CNS injury, can protect neurons against excitotoxic or ischemic lesions^102,103^ and may also modulate microglial activation and migration^104^. *Serpine1* is therefore part of the endogenous neuroprotective response to injury. We found significantly decreased gene expression of *Serpine1* in rats chronically treated with ketamine or propofol.

Glycoprotein hormones, alpha polypeptide is a protein that is encoded by the *Cga* gene. A previous study found significantly decreased *Cga* expression when mice where chronically exposed to restraint stress^105^. We found significantly decreased *Cga* gene expression when rats were chronically exposed to isoflurane, ketamine or propofol.

This study was also able to identify some unexpected genes that are more commonly known for their roles as tumour biomarker and all of which have no known role in brain or anaesthesia research. These include *Fcrl2, Asb15, Cry61, Shroom2, Tm6sf2, Defa5* and *Tshb. Fcrl2* encodes a member of the immunoglobulin receptor superfamily and is a Fc receptor-like glycoproteins located on the long arm of chromosome 1. Previously it has been suggested that this protein may be a prognostic marker for chronic lymphocytic leukemia^106^. Currently there is no research on *Fcrl2* in brain, indicating that our study may have uncovered another role for this gene. We found that acute treatment with isoflurane, ketamine and propofol significantly increased *Fcrl2* gene expression.

Ankyrin repeat and suppressor of cytokine signaling box-containing protein (ASB) 15 is a novel ASB gene expressed in skeletal muscle. Studies have shown that overexpression of *Asb15* impedes mouse myoblast differentiation suggesting activation of the Erk1/2 signaling pathway and also modifies protein turnover^107^. Research has shown that stably transfected myoblasts with decreased *Asb15* expression increased differentiation^107^. Expression of *Asb15* alters phosphorylation of the PI3K/Akt pathway, as cells with decreased *Asb15* expression have increased Akt phosphorylation^107^. *Asb15* may be important in early myoblast differentiation mediated by the PI3K/Akt signal transduction pathway^107^. Currently there is no research on *Asb15* in brain, indicating that our study may have uncovered another role for this gene. We found that acute treatment with both isoflurane and propofol significantly decreased *Asb15* gene expression.

*Cyr61* is a secreted, cysteine-rich, heparin-binding protein encoded by a growth factor-inducible immediate–early gene which acts as an extracellular, matrix-associated signaling molecule^108^. *Cyr61* stimulates the endothelial cell adhesion through an interaction with the integrin αVβ3 and augmenting growth factor-induced DNA synthesis^108^. *Cyr61* has been shown to promote angiogenesis and tumor growth^108^, be an early biomarker of renal injury^109^ and plays a role in inhibiting vascular smooth muscle cell proliferation and neointimal hyperplasia^110^. However, no previous research has shown a downregulation of *Cyr61* gene expression in brain tissue or in relation to any anaesthesia exposure. We found that acute treatment with isoflurane, propofol and ketamine and chronic treatment with isoflurane and propofol downregulated *Cyr61* expression.

*Shroom2* represents the apical protein (APX) gene, which is implicated in amiloride-sensitive sodium channel activity, is expressed in endothelial cells and facilitates the formation of a contractile network within endothelial cells. This gene is highly expressed in the retina and is a strong candidate for ocular albinism type 1 syndrome. *LOC685699/Shroom2* showed increased expression in rats treated acutely with propofol but significantly decreased in rats treated acutely with ketamine. There are no previous studies on *Shroom* expression in rat brain tissue.

*Tm6sf2* is the transmembrane 6 superfamily 2 human gene and its exact function is currently unknown. *Tm6sf2* expression was significantly increased in rats treated acutely with ketamine and propofol, however no previous studies have detailed *Tm6sf2* gene expression in the rodent brain or in anaesthesia studies.

Defensin, alpha 5 (*Defa5*) is a protein that is encoded by the DEFA5 gene and is expressed in the Paneth cells of the ileum. Defensins are a family of microbicidal and cytotoxic peptides thought to be involved in host defence. Significantly increased gene expression of *Defa5* was found in rats treated chronically with isoflurane or ketamine however no previous studies have reported *Defa5* gene expression in rat brain tissue or in relation to anaesthetic exposure.

Thyrotropin-stimulating hormone (TSH) is a noncovalently linked glycoprotein heterodimer and is part of a family of pituitary hormones containing a common alpha subunit and a unique beta subunit that confers specificity. *Tshb* was found to be significantly decreased in expression levels in rats that were chronically treated with isoflurane, ketamine or propofol. No previous studies have looked at the *Tshb* expression levels in whole rat brain with or without anaesthetic exposure.

In conclusion, from this study we can see that both chronic and acute anaesthesia exposure induces significant changes in brain biochemistry by gene expression analysis. When comparing between acute and chronic treatments we found 18 genes in control, 17 genes in isoflurane, 5 genes in ketamine and 5 genes in propofol that were significantly differentially expressed. Acute exposure to isoflurane significantly altered the expression of 27 genes when compared to control, 34 genes in ketamine treated animals and 24 in propofol exposed rats. Chronic treatment of rats with isoflurane or ketamine significantly altered 18 genes expression and propofol altered 15 genes expression levels when compared to control. A total of 11 genes were significantly altered in 2 or more acute treatments and 7 in chronic treatments. Of these, 3 were significantly different in both acute and chronic treatments. This study uncovered new genes of interest that have previously not been studied in the brain such as *Asb15, Cyr61, Fcrl2, LOC685699/Shroom2, Tm6sf2, Defa5* and *Tshb*. We were also able to confirm and support previous findings in relation to several genes such as *Arc, Egr2, Hspb1, Npas4, Nr4a3, Cga* and *Serpine1*. This study has been able to identify genes and pathways altered by anaesthesia which will allow future mechanistic studies to improve our understanding of how anaesthetics impact on neurodegeneration.

## Methods

### Animals

All animal procedures were approved by the Animal Welfare Committee of the University of Sydney (Project number 2016/1059) and performed in accordance with the National Health and Medical Research Council code of practice for care and use of animals and the NSW Animal Research Act (1985). Three-week-old male Sprague Dawley rats were obtained from Animal Resources Centre (ARC) (Perth, WA, Australia). Upon arrival they were housed in a Laboratory Animal Services (LAS) managed SPF facility at the Charles Perkins Centre and acclimatized for a period of 1 week (7 days). Procedure and holding rooms were maintained at 22 °C with a humidity of 40–60%. Rats were housed in groups of four in standard LAS provided cages (Tecniplast, GR1800 Double decker cages, 1862 cm^2^) with environmental enrichment (polycarbonate rat retreat tubes), under controlled conditions (12 h light-dark cycle) and with ad libitum access to food (standard chow – sourced from Specialty Feeds (Western Australia) Protein 14.5%, Total fat 4.8%, Total Carbohydrate 59.4%)and water. Rats were handled daily for approximately two minutes per rat for one week prior to the start of the experiment and twice weekly throughout the duration of the study. Forty-eight rats were randomized (using a random number generator) and six animals were allocated to each of the following cohorts: acute control, chronic control, acute isoflurane, chronic isoflurane, acute ketamine, chronic ketamine, acute propofol and chronic propofol.

### Anaesthetic exposure

Animals were continuously monitored for the duration of the treatment and during the recovery period. Animal body temperature was maintained at approximately 37 °C during anaesthesia using a heat pad (Harvard Apparatus, Massachusetts USA). Body weights were recorded and the appropriate dose of anaesthesia per individual animal was calculated.

Isoflurane treatment involved placing rats in a Plexiglas chamber (Harvard Apparatus, Massachusetts USA) flushed continuously with a mixture of 5% isoflurane and 95% oxygen to induce anaesthesia then continuously with a mixture of 1.2% isoflurane and 98.8% oxygen for the remaining treatment time. Isoflurane and oxygen were delivered using a calibrated flow-meter. Propofol treatment involved administration with 75 mg/kg of propofol in combination with 0.23 mg/kg of medetomidine in standard saline solution (0.9%) via IP injection. Rats were then placed in a plexiglas chamber supplied with 100% oxygen at a rate of 6 L/minute. Time till anaesthesia was measured and animals monitored over the period of an hour. Ketamine treatment involved the administration of 38 mg/kg of ketamine in combination with 0.25 mg/kg medetomidine in standard saline solution (0.9%) via IP injection. Rats were then placed in a Plexiglas chamber supplied with 100% oxygen at a rate of 6 L/minute. Time till anaesthesia was measured and animals monitored over the period of an hour. Rats in control groups (acute or chronic) were placed in a Plexiglas chamber supplied with 100% oxygen at a rate of 6 L/min delivered by a calibrated flow-meter.

When the treatment period of an hour was concluded the rats were moved to another plexiglass chamber with a heat pad for a ‘recovery period’ before being returned to their group cage under supervision. If a prolonged recovery period was experienced the animal was administered with a standard saline solution (0.9%) (Team Medical supplies, NSW, Australia) injection to prevent dehydration.

The acute exposure group underwent a single 1-hour treatment at 4 months of age before the study termination and the chronic exposure group underwent three 1-hour treatments at the age of 1, 2 and 4 months old before study termination.

### Study termination

Rats were decapitated by guillotine (World Precision Instruments, Florida, USA) (immediately post final treatment) either under anaesthesia (for isoflurane, ketamine and propofol treatment groups) or without anaesthesia (control group) at the end of the final chronic treatment or post-acute treatment at 4 months of age. Whole brains were removed and snap frozen in liquid nitrogen before being transferred to −80 °C for storage and later use for microarray analysis.

### RNA isolation and purification

Total RNA was extracted from the whole brains using standard TRIzol extraction methods. Briefly, whole brain samples were transferred from −80 °C storage and quickly homogenized using the Qiagen TissueRuptor (Qiagen, VIC, Australia) in 15 mL of TRIzol (Sigma-Aldrich Pty. Ltd., NSW, Australia). Samples were then aliquoted into ten 1.5 mL eppendorf tubes (Eppendorf South Pacific PTY Ltd, NSW, Australia) and stored at −80 °C, leaving one for further RNA processing. Chloroform (Sigma-Aldrich Pty. Ltd., NSW, Australia) was added and samples were centrifuged at 12,000 g for 15 minutes allowing for clean phase separation to occur. The upper aqueous phase was transferred to a new tube and 100% isopropanol (Sigma-Aldrich Pty. Ltd., NSW, Australia) added before overnight incubation at −80 °C. The following day samples were incubated at room temperature for 10 minutes before centrifugation at 12,000 g for 10 minutes. The RNA pellet was then washed with 75% ethanol before being re-suspended in RNA-free water (Life Technologies Pty. Ltd., VIC, Australia) and stored at −80 °C. RNA purification was undertaken using the Qiagen midi-prep kit (Qiagen, VIC, Australia) (following manufacturer’s instructions) and resulting RNA quantified on a ThermoFisher Nanodrop (Thermo Fisher Scientific, NSW, Australia) and the RNA integrity number (RIN) verified using an Agilent 4200 TapeStation Bioanalyzer (Agilent Technologies Australia, VIC, Australia).

### RNA analysis

Whole brain RNA from control and anaesthetic treated rats was adjusted to a concentration of 100 ng/uL, samples randomized and blinded and sent to the Ramaciotti Centre for Genomics at University of New South Wales (UNSW) where RNA analysis was undertaken. At UNSW the samples underwent hybridization to Affymetrix Clariom S microarrays (Thermo Fisher Scientific, NSW, Australia). cDNA synthesis, *in vitro* transcription, cRNA fragmentation, and hybridization reactions were all carried out as recommended by Affymetrix. Detection of changes for each change were made by the Affymetrix Microarray Suite version 5.0 software based on the hybridization signals produced by the matched oligos.

### Gene expression analysis

Affymetrix Expression Console was used to process the original.CEL files using Clariom_S_Rat library file from Affymetrix. The.chp files were generated using the RMA-sketch workflow after signal summarization (Median polish) and data normalization (Sketch-Quantile method). Gene level analysis was further conducted with Affymetrix Transcriptome Analysis Console 2.0 software. A total of 22,000 genes and 129,800 transcripts were tested at core level to compare their expression between the control and anaesthetic treated groups. Scatter plot bar graphs have been provided in Supplementary Figs. 1 and 2 for the signal intensity of the genes in Figs. 3 and 4. All cohorts included 6 replicates except for control which only had 5 replicates.

### Statistical analysis

Brain weights were compared by one-way ANOVA between control and treatment groups.

To understand the effects of different anesthetic treatments on brain gene expression, differential expression analysis of the microarray data was performed using multiple stringency levels. The first stringency threshold of *p* < 0.05 was used to visualize the changes that accompany each treatment and is independent of expression magnitude. The second threshold of *p* < 0.05 with |fold change | > 2 was used to identify the specific genes that are differentially expressed in each treatment group (isoflurane, ketamine, propofol) or treatment frequency (acute, chronic). Probesets were only considered expressed if >50% of samples had values below DABG (detection above background) threshold. Using this 2 fold change in gene expression as our threshold permitted an experimental variance of 0.7 to detect a true difference with a sample size n = 6, power 0.80, and Type 1 error probability α = 0.05. According to the algorithm of Affymetrix Transcriptome Analysis Console 2.0, ANOVA was applied to calculate the p-value. ANOVA analysis using the limma function eBayes was used to calculate the gene-wise statistics for all microarray data. The limma function enables samples to be allocated an attribute which can be comparison, repeated measure, batch effect, random factor, real covariate and none. For our analysis, comparison type was selected which allows differential gene expression analysis and interactions between the different attributes to be computed. The eBayes analysis corrects the variance of the ANOVA analysis with an empirical Bayes approach that uses the information from all the probesets to yield an improved estimate for the variance. The eBayes correction is especially important when the number of samples being analyzed is small.

## Supplementary information

Supplementary information.

Supplementary information2.

Supplementary information3.

## Data Availability

The datasets generated during and/or analysed during the current study are available from the corresponding author on reasonable request.
