# Supplementary material for: Anaesthetic-dependent changes in gene expression following acute and chronic exposure in the rodent brain
Source: Sci Rep. 2020 Jun 9;10:9366. doi: 10.1038/s41598-020-66122-6 (PMC7283325; doi:10.1038/s41598-020-66122-6)
Supplement: Supplementary file 1 — Supplementary information. [file 41598_2020_66122_MOESM1_ESM.pdf]

Anaesthetic-dependent changes in gene expression following acute and chronic exposure in the rodent brain

Dannielle H. Upton, Kata Popovic, Roger Fulton, and Michael Kassiou

Gene Symbol	Acute Avg (log2)	Chronic Avg (log2)	Fold Change	ANOVA p-value
**Control**				
Tph1	4.67	12.68	-258.71	0.0259
Pde6g	4.68	7.29	-6.12	0.0142
Neurod4	4.2	6.46	-4.8	0.0191
Gnat2	4.43	6.1	-3.19	0.0368
Pde6c	4.98	6.53	-2.94	0.0042
Gngt1	5.07	6.33	-2.4	0.0008
Hoxa5	3.32	4.55	-2.34	0.003
Hoxa6	3.55	4.65	-2.14	0.002
Tnfaip2	5.22	6.29	-2.09	0.0049
Ighg; Igh-6	5.72	6.78	-2.08	0.0091
Guca1a	3.48	4.52	-2.05	0.0191
Vom1r50	4.99	5.99	-2	0.0325
Vom2r1	4.12	3.11	2.01	0.001
Cyp4v3	9.05	8.04	2.02	0.0036
Olr1700	4.1	3.02	2.11	0.0123
Tshb	6.6	5.5	2.15	0.0423
Slamf1	5	3.85	2.22	0.0015
Fcrl2	6.7	4.69	4.01	0.009
**Isoflurane**				
Plekhh3	5.32	6.51	-2.28	0.0038
Nuak2	3.41	4.58	-2.25	0.0009
Ttc30a	3.81	4.98	-2.25	3.78E-05
XAF1	4.76	5.83	-2.09	0.006
C3	6.13	7.14	-2.02	0.0189
LOC102555445	5.61	4.61	2	0.0467
Ccl17	3.56	2.55	2.01	0.032
Itpripl1	6.91	5.88	2.04	0.0164
Lmod2	3.87	2.84	2.05	0.0223
Pi15	5.39	4.35	2.06	0.012
Gdf9	4.83	3.75	2.11	0.0374
Csmd2	7.58	6.51	2.11	0.0034
Grifin	5.56	4.45	2.17	0.0113
Frmd7	4.16	3	2.24	0.0455
Wdr49	5.51	4.3	2.32	0.0027
RT1-M6-2	5.31	3.79	2.86	0.0199
Asb15	11.52	3.58	246.3	4.22E-05
**Ketamine**				
Sycp2l	4.43	5.69	-2.4	0.0405
Avp	13	14.14	-2.2	0.0093
Mcm5	3.62	4.72	-2.14	0.0048
Bpifb3	3.74	4.81	-2.1	0.0069

Inca1	3.7	4.73	-2.04	0.0013
Vom2r1	4.32	3.27	2.07	0.0066
Olr586	4.41	3.3	2.15	0.0064
Ifna16l1	5.15	3.92	2.34	1.66E-06
Ly49s7; Klra5	4.25	3.01	2.35	0.0247
**Propofol**				
Hoxa6	3.46	4.56	-2.14	0.0342
Zfp72	5.41	6.5	-2.12	0.0473
LOC102553524	5.75	6.82	-2.09	0.0012
Bmp4	9.87	8.84	2.04	0.0215
Klkb1	4.64	3.34	2.47	0.0107

Supplementary Table 1 Acute treatments vs Chronic treatments - Detailed average expression levels, fold change and p-values for significant probe sets. Non functionally characterised genes have been removed from analysis

Gene Symbol	Acute Control Avg (log2)	Acute Treatment Avg (log2)	Fold Change	ANOVA p-value
**Isoflurane**				
Asb15	3.8	11.65	-230.12	0.0001
Tshb	6.02	7.38	-2.57	0.0002
Rdh5	5.98	7.17	-2.28	0.019
She	5.23	6.35	-2.17	0.0012
LOC100360997	4.9	6.01	-2.15	0.0047
Sox18	5.24	6.32	-2.11	0.0004
LOC100911790	3.89	4.92	-2.04	0.0256
Aox1	4.8	5.83	-2.03	0.0291
Spin2a	3.68	4.7	-2.02	0.0021
Cyr61	5.18	6.19	-2.01	0.0286
Wdr49	4.62	5.63	-2.01	0.0023
Ccnd3	5.23	4.22	2	1.75E-05
Nr4a3	9.87	8.81	2.09	0.0124
Dusp1	14.79	13.73	2.1	0.0183
Pdk4	7.21	6.14	2.1	0.0085
Olr1055	4.24	3.17	2.1	0.0121
Nr4a1	13.53	12.45	2.12	0.0257
RGD1560527	4.93	3.84	2.14	0.0028
Hspb1	5.97	4.87	2.14	0.0183
RGD1560821	5.4	4.24	2.23	0.0013
Nfkbia	9.99	8.82	2.26	0.0026
Egr2	8.62	7.38	2.35	0.0095
Npas4	9.44	8.09	2.55	2.84E-05
NONMMUG006795	4.72	3.31	2.65	0.0208
Rpl39l	4.18	2.54	3.12	0.0164
Arc	10.36	8.61	3.36	0.0005
Fcrl2	5.94	3.66	4.86	2.83E-05
**Ketamine**				
Tph1	4.99	11.96	-125.39	0.0336
Cyr61	5.53	6.94	-2.67	0.0038
Vom2r25	2.94	4.3	-2.57	0.0004
LOC680910	5.88	7.2	-2.5	0.0076
LOC685699	4.45	5.75	-2.47	0.0018
Ly49s7; Klra5; LOC100911234	3.46	4.66	-2.29	0.0068
Apold1	6.44	7.63	-2.27	7.90E-05
Olr59	8.04	9.21	-2.24	0.0309
Igkc; LOC500183	5.81	6.97	-2.24	0.0259
Klkb1	3.56	4.72	-2.24	0.0016
Dkk2	4.16	5.32	-2.23	0.0009
Ly49i7; LOC100911272	3.5	4.65	-2.22	0.0354
Casz1	4.89	5.99	-2.15	0.0006
Ahsp	3.93	5.01	-2.12	0.0003
Fign	4.87	5.93	-2.09	0.0078
Ifit3	4.64	5.69	-2.07	0.0009
Foxf1	6.21	7.23	-2.03	0.0026

NONMMUG036691	3.24	4.25	-2.01	0.0301
LOC307727	3.53	2.52	2.02	0.0036
Wfikkn1	6.31	5.28	2.04	0.0066
LOC100912354	5.47	4.42	2.06	0.0076
Mboat4	5.17	4.11	2.08	0.0001
Nr4a1	13.57	12.51	2.09	0.0003
LOC502908	5.62	4.53	2.13	0.0349
Gdf9	5.06	3.96	2.14	0.0422
Tm6sf2	5.84	4.65	2.29	0.0011
Pdzd3	4.65	3.45	2.29	0.0004
Ang; Rnase4	12.09	10.85	2.37	0.0265
Egr2	8.93	7.63	2.46	0.0026
Fos	10.04	8.69	2.55	0.001
Npas4	9.65	8.23	2.69	3.50E-06
Slc39a12	8.39	6.87	2.88	0.0205
Arc	10.72	8.59	4.38	6.90E-07
Fcrl2	6.57	4.32	4.75	6.70E-05
**Propofol**				
Asb15	3.96	11.98	-259.24	0.0102
Cyr61	5.56	6.82	-2.4	0.0003
Nt5c3a	12.24	13.45	-2.33	0.0005
Klkb1	3.23	4.41	-2.27	0.0008
Rbp3	3.26	4.4	-2.19	0.0183
Ighg; Igh-6	5.89	6.99	-2.15	0.0182
LOC685699	4.24	5.34	-2.15	0.0158
Scgn	5.05	6.11	-2.08	0.0002
Tor4a	5.84	6.87	-2.03	0.022
Vwa3b	4.05	5.06	-2.01	0.0019
Nr4a3	10.07	9.07	2	0.0019
Pax5	4.01	3	2.02	0.0034
Nr4a1	13.75	12.72	2.05	0.0005
LOC499219	4.71	3.65	2.09	0.0412
Olr1539; Olr1537	4.81	3.72	2.14	0.0048
Hspb1	6.64	5.49	2.22	0.0038
Il13ra2	5.22	4.04	2.26	0.0071
LOC100361731	3.81	2.6	2.31	0.0242
Tm6sf2	5.94	4.67	2.41	4.36E-05
Egr2	8.9	7.52	2.6	0.002
Npas4	9.77	7.91	3.62	3.89E-07
Arc	10.89	9.02	3.65	9.87E-06
Fcrl2	6.87	4.97	3.75	0.0377
Zfp72	7.27	4.66	6.13	0.0229

Supplementary Table 2 Acute Control vs Acute Treatments - Detailed average expression levels, fold change and p-values for significant probe sets. Non functionally characterised genes have been removed from analysis

Gene Symbol	Chronic Control Avg (log2)	Chronic Isoflurane Avg (log2)	Fold Change	ANOVA p-value
**Isoflurane**				
Cga	7.22	9.35	-4.37	0.0015
Tshb	5.04	7.16	-4.35	0.0022
Bmp4	8.78	10.26	-2.78	0.0324
Cyr61	5.41	6.84	-2.7	0.0004
Hes5	6.34	7.64	-2.46	0.0002
Id1	10.82	11.94	-2.17	0.0001
Hes1	7.67	8.73	-2.08	0.0001
Olr1319; Olr1320; Olr1323; Olr1325; Olr1327; Olr1328; Olr1321; Olr1318	3.06	4.12	-2.07	0.0001
Spetex-2E	5.08	6.09	-2.02	0.0377
Defa5	5.1	4.08	2.02	0.001
Nfkbia	10.02	8.99	2.05	0.0024
Fos	9.46	8.42	2.06	0.0289
RGD1562035	5.03	3.96	2.09	0.0067
ENSMUSG00000098857	6.28	5.15	2.18	0.016
Gngt1	6.9	5.75	2.22	0.0176
Npas4	9.49	8.23	2.39	8.25E-06
LOC102555445	6.24	4.8	2.71	0.0403
LOC100909947	7.08	5.43	3.14	0.0414
**Ketamine**				
Tshb	4.96	7.09	-4.39	0.0009
Cga	7.29	8.99	-3.26	0.0019
Hoxa2	4.18	5.5	-2.5	0.0194
Serpine1	3.93	5.18	-2.39	7.41E-05
Foxf1	6.17	7.4	-2.35	8.15E-05
Apold1	7	8.1	-2.14	0.0009
LOC500300	4.31	5.4	-2.13	0.0007
Aurkb	4.1	5.17	-2.09	0.0347
LOC691196	3.33	4.36	-2.04	0.0083
Ifit3	4.93	5.94	-2.02	0.0004
Atf3	4.41	5.42	-2.01	2.05E-05
Hoxc4	6.54	5.47	2.11	0.0002
Olr802	4.46	3.38	2.12	0.022
LOC100912565	4.65	3.52	2.19	1.18E-05
Defa5	5.18	3.99	2.29	0.0012
LOC308990	5.08	3.84	2.35	0.0032
Arc	10.03	8.57	2.74	0.0002
Npas4	9.8	8.2	3.03	4.24E-07
**Propofol**				
Asb15	4.17	8.42	-19.01	0.0109
Tshb	5.28	7.04	-3.36	0.001
Cga	7.36	9.03	-3.18	0.0038
Serpine1	4.17	5.48	-2.48	3.45E-05

LOC500300	3.92	5.08	-2.23	0.0401
Cyr61	5.46	6.58	-2.18	0.0001
Pate-f; LOC100911894	3.82	4.94	-2.17	0.0191
Lrrc39	4.23	5.33	-2.14	0.0013
Lrrc32	6.4	7.41	-2.02	0.0012
Junb	10.53	9.51	2.03	0.0022
LOC102553881; LOC102553382	8.67	7.62	2.06	0.001
LOC685289; LOC102553520; LOC685275	6.43	5.38	2.08	0.0079
Dusp12	10.52	9.42	2.14	0.0122
Arc	10.05	8.84	2.32	0.0024
Npas4	9.88	7.64	4.7	2.62E-07

Supplementary Table 3 Chronic Control vs Chronic Treatments - Detailed average expression levels, fold change and p-values for significant probe sets. Non functionally characterised genes have been removed from analysis
