# Supplementary figures and images for: Anaesthetic-dependent changes in gene expression following acute and chronic exposure in the rodent brain
Source: Sci Rep. 2020 Jun 9;10:9366. doi: 10.1038/s41598-020-66122-6 (PMC7283325; doi:10.1038/s41598-020-66122-6)

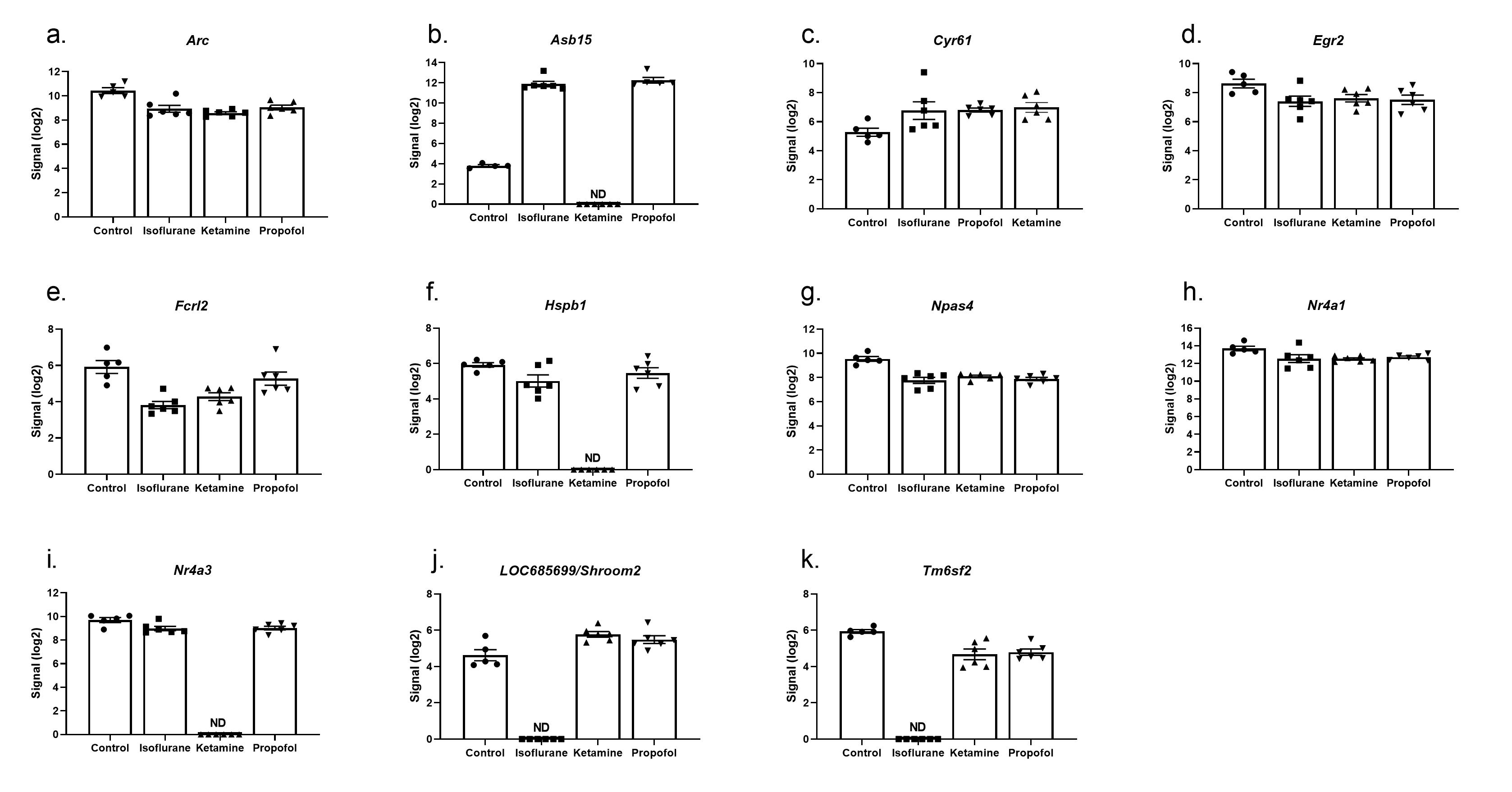

Supplement: Supplementary file 2 — Supplementary information2. [file 41598_2020_66122_MOESM2_ESM.jpg]

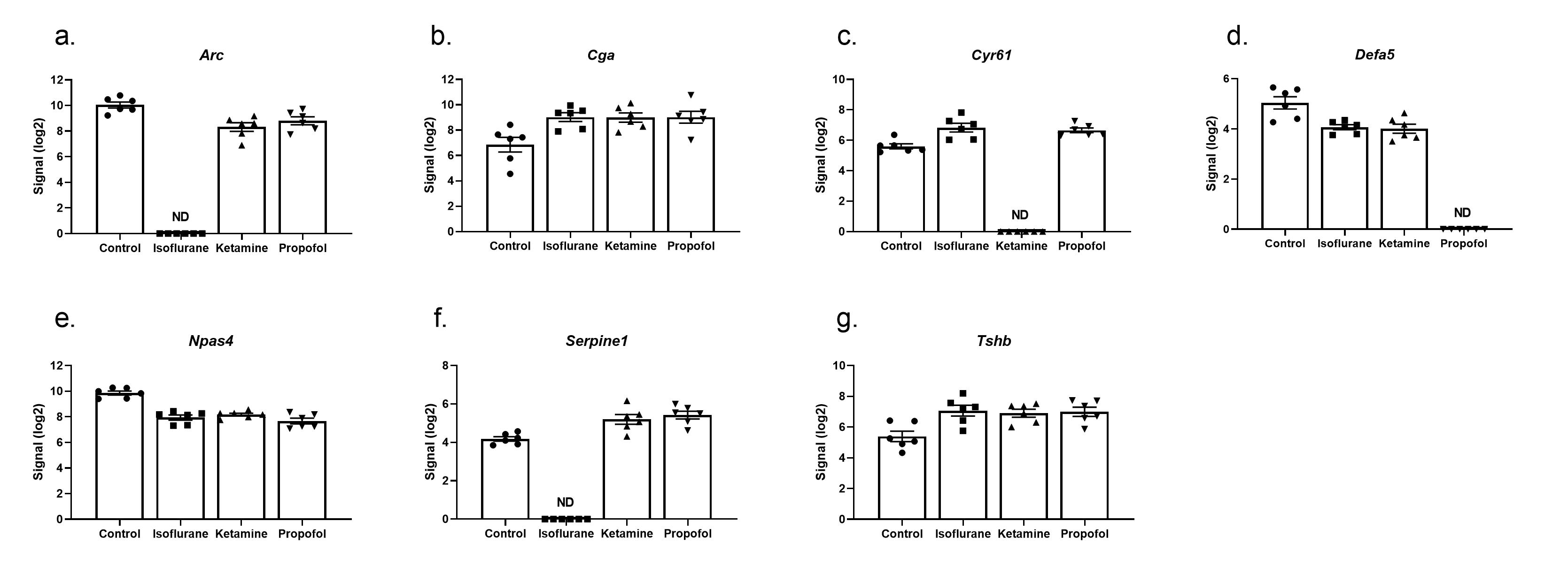

Supplement: Supplementary file 3 — Supplementary information3. [file 41598_2020_66122_MOESM3_ESM.jpg]
